# Natural vs. artificial cannabinoid oils: the comparison of their antioxidant activities

**DOI:** 10.1007/s00217-022-04121-9

**Published:** 2022-09-22

**Authors:** Andrzej L. Dawidowicz, Rafał Typek, Małgorzata Olszowy-Tomczyk

**Affiliations:** Department of Chromatography, Institute of Chemical Sciences, Faculty of Chemistry, Maria Curie-Sklodowska University in Lublin, Pl. Marii Curie Sklodowskiej 3, 20-031 Lublin, Poland

**Keywords:** Cannabinoid oils, Antioxidant activity of cannabinoid oils, CBG oil, CBD oil, THC oil

## Abstract

In the wide range of products containing hemp ingredients, cannabinoid oils are the most popular. They have gained popularity not only among people struggling with various health ailments, but also those who search for a neutral way of taking care of their body and mind. The antioxidant activities of cannabinoid oils differing in the type of their main cannabinoid [i.e., Cannabigerol (CBG), Cannabidiol (CBD), Δ9-Tetrahydrocannabinol (Δ9-THC), Cannabigerolic acid (CBGA), Cannabidiolic acid (CBDA) or Δ9-Tetrahydrocannabinolic acid (Δ9-THCA)] are compared and discussed in the paper. The oils with the same concentration of their main cannabinoid but prepared in different ways were applied in the experiments. Following the presented results, cannabinoid oils obtained from the plant extracts are characterized by evidently greater antioxidant activity than those prepared from pure cannabinoids. The essential difference in the antioxidant activity of the oils containing the neutral or acidic form of a given cannabinoid is observed only in the case of THC and THCA oils.

## Introduction

Positive effect of some cannabinoids in the treatment and prophylaxis of a wide variety of oxidation-associated diseases results in a growing interest in different products, particularly in dietary supplements containing ingredients of hemp sativa and marijuana [1–6]. In a fairly wide range of such products available on the market [7, 8], cannabinoid oils (e.g., CBD oil, CBG oil) have been enjoying great popularity recently. In the group of commonly used cannabinoid oils, there can be distinguished two types of the oils—those prepared from pure cannabinoids and those obtained from plant extracts. In the metabolic pathway of hemp plant, the precursors of neutral cannabinoids are their acidic forms. Thus, raw hemp extracts contain mainly acidic forms of cannabinoids. As biological activities of the neutral and acidic forms of cannabinoids are somewhat different, two types of cannabinoid oils prepared from the plant extracts are delivered to the market—so-called raw cannabinoid oils containing mainly acidic forms of cannabinoids and decarboxylated cannabinoid oils containing mainly neutral cannabinoids. It is worth noting that cannabinoid oils prepared from the plant extracts are complex mixtures. In addition to cannabinoids, the main antioxidant agents in cannabinoid oils, they contain a number of compounds isolated from the plant matrix. Apart from chlorophylls and waxes, they include also terpenes and polyphenolic compounds which also exhibit the antioxidant activity [9–13]. The antioxidant activities of different cannabinoid oils obtained from the plant extracts and from pure cannabinoids were compared and discussed in the paper. To determine the influence of other hemp matrix components on the antioxidant activity of cannabinoid oils, the cannabinoid oils, both those prepared from the plant extracts and from the pure cannabinoids, of the same concentration of the dominating cannabinoids were employed in the experiments. To estimate the antioxidant activity of the prepared oils, the 2,2'-azino-bis(3-ethylbenzothiazoline-6-sulfonic acid (ABTS) assay was used. This method is regarded as a direct, rapid, simple and reliable for the estimation of the antioxidant activity of the cannabinoid oils being examined as the concentration changes of the ABTS cation radical are registered spectrophotometrically at the 744 nm wavelength which does not coincide with the spectrum of the tested cannabinoids and their oxidation products [14, 15]. Hence, the concentration changes of cannabinoids and their derivatives in the measuring system do not influence on the reliability of measured antioxidant activity [16, 17].

## Materials and methods

### Materials

Methanol (pure analytical), acetonitrile (HPLC grade) and dichloromethane were supplied by the Polish Chemical Plant POCh (Gliwice, Poland). CBD, CBG, Δ9-THC, CBDA, CBGA and Δ9-THCA, 2,2′-azinobis(3-ethylbenzothiazoline-6-sulfonic acid) di-ammonium salt (ABTS), di-potassium peroxydisulfate and formic acid were purchased from Sigma Aldrich (Poznań, Poland). Deionized water was purified by means of a Milli-Q system from Millipore (Millipore, Bedford, MA, USA). Hemp plants, Santhica 27, Futura 75 and Indica Kush, were the gifts from local growers from the district of Lublin (Poland). Commercially available hemp oil commercially distributed with a healthy food was used as a base of the examined cannabinoid oils.

### Hemp extracts preparation

10 g of dried and milled hemp plant (Santhica 27 or Futura 75 or Indica Kush) was macerated with 100 ml of dichloromethane in a glass Erlenmeyer flask for 45 min at 35 °C. The vessel with the suspension of the given hemp plant was placed in the ultrasonic bath (Sonic-6, Polsonic, Poland) working with 40 kHz frequency and 540 W power. After this process, the liquid phase was separated on the Buchner funnel and concentrated using CentriVap Bentchtop Vacuum Concentrator (Labconco, USA) at 35 °C.

Each of the obtained extracts was divided into two parts. The first one, not additionally treated, was used directly to prepare the cannabinoid oils rich in acidic forms of cannabinoids, whereas the second one was heated in the SLN 15 laboratory dryer (POL-EKO, Poland) for 45 min at 140 °C to transform acidic forms of cannabinoids into neutral ones. These decarboxylated extracts were used to prepare the cannabinoid oils rich in neutral forms of cannabinoids. Before cannabinoid oils preparation, all hemp extracts were examined by High-Performance Liquid Chromatography (HPLC) with respect to the presence and concentration of their 6 main cannabinoids, i.e., CBGA, CBDA, THCA, CBG, CBD, THC. The concentrations of these cannabinoids in individual extracts are presented in Table 1. The concentration of the main cannabinoid in the given plant extract is bolded.

**Table 1:** Concentration of CBGA, CBDA, THCA, CBG, CBD and THC in Santica 27, Futura 75 and Indica Kush extracts before and after their thermal treatment

Hemp type	Thermal treatment of hemp extract	Concentration in [%] of:					
		CBGA	CBG	CBDA	CBD	THCA	THC
Santhica 27	No	**37.71**	1.55	4.73	0.28	0.72	0.03
	Yes	0.41	**33.63**	0.07	4.24	0.01	0.64
Futura 75	No	0.52	0.03	**35.22**	1.86	0.43	0.03
	Yes	0.01	0.51	0.29	**31.51**	0.01	0.38
Indica kush	No	1.00	0.04	4.40	0.19	**39.41**	2.66
	Yes	0.04	0.81	0.07	3.85	0.46	**35.05**

### Preparation of cannabinoid oils

Three types of cannabinoid oils were prepared for these research.

The first group were oils obtained with the use of hemp plant extracts. Therefore, they are referred to as natural (NAT) cannabinoid oils. For their preparation, these were used as raw extracts from individual plants which contained mainly acidic forms of cannabinoids as well as decarboxylated extracts (those obtained by a proper thermal treatment of raw plant extracts) which contained mainly neutral forms of cannabinoids. The names of individual NAT oils contain the short name of the cannabinoid that dominates in them—e.g., CBGA-NAT refers to the oil obtained by dissolving the raw Santhica extract (CBGA is the major cannabinoid in this extract) in hemp oil and, e.g., CBD-NAT means the oil obtained by dissolving the decarboxylated Futura extract (CBD is the major cannabinoid in this extract) in hemp oil.

The concentrations of the main cannabinoid in all NAT oils were the same being 0.353 M. The percent concentrations of the individual cannabinoids in the prepared NAT oils under examination and estimated by HPLC are given in Table 2. As the table shows, the obtained oils are analogous with those commercially available containing about 10% of the main cannabinoid.

**Table 2:** Concentration of CBGA, CBDA, THCA, CBG, CBD and THC in NAT and ART oils and in the methanolic solutions of hemp plant extracts

Cannabinoid	Cannabinoid concentration [%] in oil or methanolic solution of hemp extract					
	CBGA-NAT or CBGA-ART or CBGA-MONO	CBDA-NAT or CBDA-ART or CBDA-MONO	THCA-NAT or THCA-ART or THCA-MONO	CBG-NAT or CBG-ART or CBG-MONO	CBD-NAT or CBD-ART or CBD-MONO	THC-NAT or THC-ART or THC-MONO
CBG	0.45	0.01	0.01	9.92*	0.16	0.23
CBD	0.08	0.57	0.05	1.25	9.94*	1.09
THC	0.01	0.01	0.72	0.19	0.12	9.93*
CBGA	10.93*	0.16	0.27	0.12	< LOD	0.01
CBDA	1.37	10.77*	1.19	0.02	0.09	0.02
THCA	0.21	0.13	10.65*	< LOD	< LOD	0.13

The concentration of a single cannabinoid in MONO oil is marked with a star

The oils of the latter group were obtained by dissolution of pure cannabinoids in hemp oil. Thus, they are referred to as artificial (ART) cannabinoid oils. 6 cannabinoids, those present in the highest concentration in individual plant extracts, were used for their preparation. The concentrations of individual cannabinoids in ART oils were the same as in their NAT counterparts, and the names of ART cannabinoid oils are analogous to those as for NAT ones—see Table 2.

The third group were the oils obtained dissolving one of the mentioned six cannabinoids in hemp oil. Thus, they are referred to as mono-component (MONO) cannabinoid oils. The concentration of a given cannabinoid in a given MONO oil was the same as that of the main cannabinoid in its NAT and ART counterpart oils—see the values with stars in Table 2. Mono-component cannabinoid oils are marked in the same way as NAT and ART oils—i.e., CBD-MONO refers to the oil obtained by dissolving CBD standard in hemp oil.

After the preparation, all ART and MONO oils were also examined by HPLC with respect to the concentration of CBGA, CBDA, THCA, CBG, CBD and THC.

Methanolic solutions of all obtained plant extracts were a separate research group. Therefore, they are related to (NAT) solutions. The names of individual MeOH solutions contain the brief name of the cannabinoid that dominates in them—e.g., CBGA–MeOH refers to the methanolic solution obtained by dissolving a raw Santhica extract (CBGA is the major cannabinoid in this extract) in methanol. The main cannabinoid concentration in all MeOH solutions was the same as in the case of NAT cannabinoid oils and amounted to 0.353 M.

### Methods

#### ABTS method

The antioxidant abilities of the examined cannabinoid oils and TROLOX were estimated spectrophotometrically registering the concentration change of the ABTS radicals at 744 nm. To ABTS cation radical formation, 7 mM aqueous ABTS solution (5 mL) and 140 mM potassium persulfate (K_2_S_2_O_8_) (88 μL) were used. After its 20 h incubation in the dark, it was diluted with methanol to the absorbance equal 0.7 ± 0.05 measured at 744 nm. The details of ABTS solution preparation can be found in the paper by Olszowy and Dawidowicz [18].

The obtained ABTS^●+^ solution (2900 µL) was mixed with the methanolic solution of the cannabinoid oil or hemp extract or TROLOX or hemp oil (100 µL) being examined in a 4 mL test tube and after shaking put into an optical glass cuvette (1× 1 × 3.5 cm) which was immediately placed in a spectrophotometer. The absorbance decrease was monitored in a continuous manner for 60 min using UV Probe-2550 Spectrophotometer (Shimadzu, Kyoto, Japan). Pure methanol was used to zero the spectrophotometer.

The inhibition percent of ABTS^●+^ was calculated according to the following equation:

$${\text{I}}\,\,\left( {\text{\% }} \right) = \left( {1 - \frac{{{\text{A}}_{{{60}}} }}{{{\text{A}}_{{0}} }}} \right) \cdot {\text{100\% }}$$

where: A_0_ and A_60_ are the values of ABTS^●+^ absorbance at 0 and 60 min of the radical neutralization reaction, respectively.

### HPLC analysis

The concentrations of CBGA, CBDA, THCA, CBG, CBD and THC in the obtained hemp extracts and cannabinoid oils were estimated by HPLC. For this purpose, Nexera-i LC-2040C 3D system with PDA detection working at 230 nm (Shimadzu, Japan) was employed. The chromatographic separations were performed at 55 °C using the SYNERGI 4u Polar-RP column (250 × 4.6 mm, 5 μm) and the Phenomenex Security Guard ULTRA LC type guard column (both from Phenomenex, USA). 5 μL of samples was injected. The mobile phase was composed of ACN with 0.1% HCOOH and water with 0.1% HCOOH (65/35 v/v). The total run time was 15 min at the 1 mL/min mobile phase flow rate.

### Statistical analysis

All results are presented as the mean value of five independent measurements (*n* = 5) ± SD. The antioxidant activities were compared using the analysis of variance (ANOVA). Antioxidant activity differences in the studied groups were considered as significant for *p* ≤ 0.05 and *F*_crit_ < *F*_exp_.

## Results and discussion

**Fig. 1:**
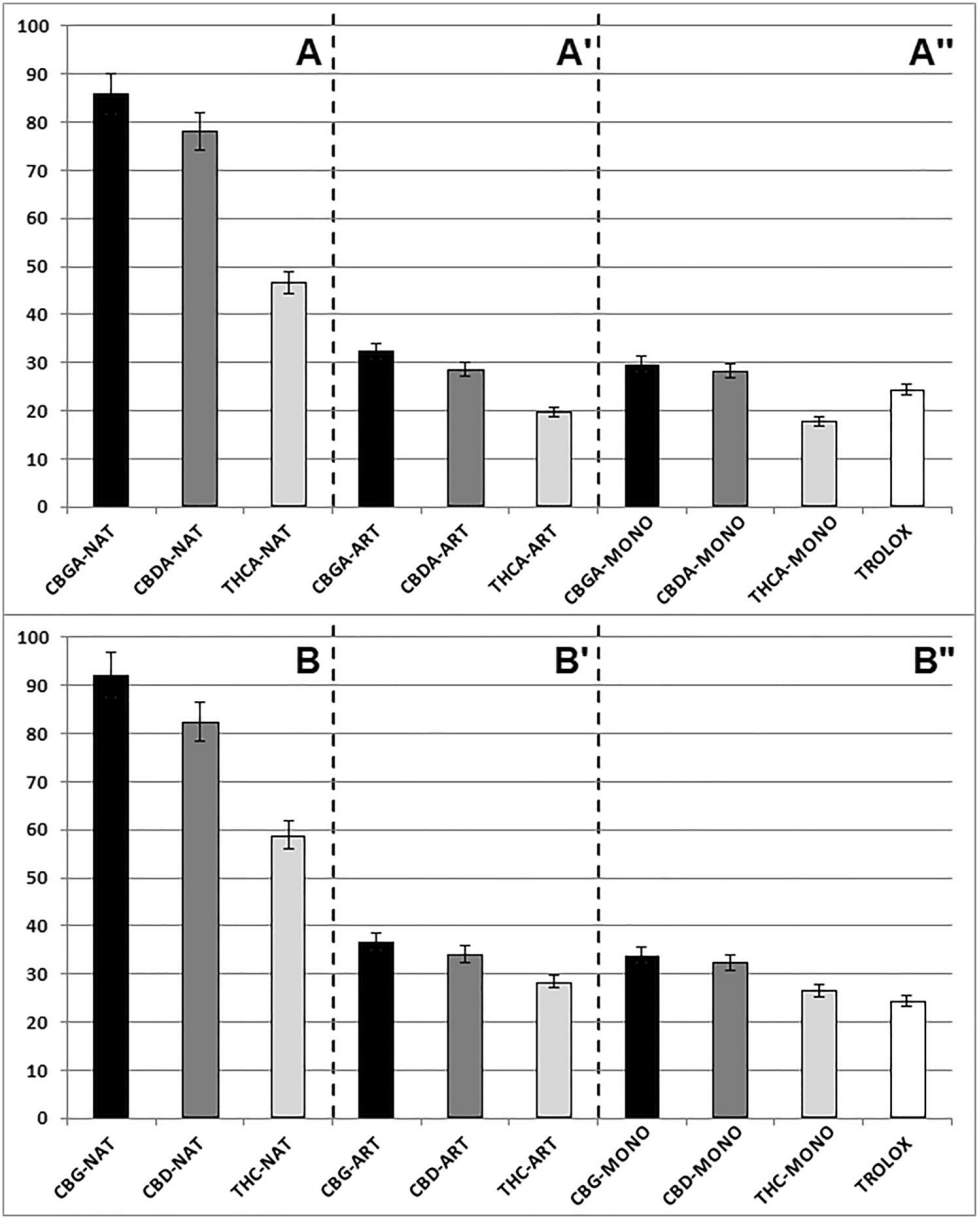
Antioxidant activity of: CBGA-NAT, CBGA-ART and CBGA-MONO cannabinoid oils (black bars in **A**, **A**’ and **A**’’, respectively); CBDA-NAT, CBDA-ART and CBDA-MONO cannabinoid oils (dark gray bars in **A**, **A**’ and **A**’’, respectively); THCA-NAT, THCA-ART and THCA-MONO cannabinoid oils (light gray bars in **A**, **A**’ and **A**’’, respectively); CBG-NAT, CBG-ART and CBG-MONO cannabinoid oils (black bars in **B**, **B**’ and **B**’’, respectively); CBD-NAT, CBD-ART and CBD-MONO cannabinoid oils (dark gray bars in **B**, **B**’ and **B**’’, respectively); THC-NAT, THC-ART and THC-MONO cannabinoid oils (light gray bars in **B**, **B**’ and **B**’’, respectively); The last white bar in **A**’’ and **B**’’ represents the TROLOX antioxidant activity

Figure 1 compares the antioxidant activities of NAT, ART and MONO cannabinoid oils containing acidic (Fig. 1A, A’ and A’’, respectively) and neutral (Fig. 1B, B’ and B’’, respectively) forms of cannabinoids. For better assessment of the antioxidant activity of individual oils, the diagrams also contain the data for TROLOX, an analog of vitamin E, which is a known antioxidant used in biological and biochemical applications to reduce oxidative stress or damage (see the last bar in Fig. 1A’’and B’’). It should be emphasized that the concentrations of the cannabinoid dominating in a given oil (e.g., CBG in CBG-NAT or CBG-ART or CBG-MONO; THCA in THCA-NAT or THCA-ART or THCA-MONO etc.) and of TROLOX in the measuring systems with the ABTS radical cation were the same and amounted to 0.0001 M.

The obtained results prove that:

- All examined cannabinoid oils, except THCA-ART and THCA-MONO oils, exhibit a greater antioxidant activity than TROLOX [*F*_exp_ > *F*_crit_; individual (*F*_exp_) values are collected in section A of Table 3]. The low activity of these two oils is connected with a smaller antioxidant activity of their main cannabinoid, Δ9-THCA, possessing only one phenolic OH group, which is additionally engaged in the formation of the hydrogen bond with the COOH group [19];
- The antioxidant activity of individual NAT cannabinoid oils is 2–3 times greater than that of their ART and MONO counterparts obtained from pure ingredients. The antioxidant activity of ART and MONO counterparts is similar [*F*_exp_ < *F*_crit_; individual (*F*_exp_) values are collected in section B of Table 3]. As the concentration of the dominant cannabinoid in the corresponding cannabinoid oils is the same (i.e., CBG in CBG-NAT or CBG-ART or CBG-MONO etc.), a significantly greater antioxidant activity of NAT oils compared to that of ART and MONO oils [*F*_exp_ > *F*_crit_; individual (*F*_exp_) values are collected in section C of Table 3] results most likely from the presence of other hemp components in the former. It should be noted that besides the main six cannabinoids, NAT oils under examination contain also trace amounts of other cannabinoids [e.g., Cannabidivarin (CBDV), Cannabinol (CBN), Cannabichromene (CBC) and others in the concentrations difficult to quantify] and non-cannabinoid plant matrix components, mainly polyphenolic compounds, which also exhibit strong antioxidant activity. Thus, the much greater antioxidant activity of cannabinoid oils obtained from the plant extracts compared to their ART and MONO counterparts obtained from pure ingredients is understandable;
- A decrease in the antioxidant activity of CBGA, CBDA and THCA oils as well as CBG, CBD and THC [*F*_exp_ > *F*_crit_; individual (*F*_exp_) values are collected in section D of Table 3] ones in distending order, both those obtained from the hemp extracts (NAT) and from the pure ingredients (ART and MONO), is observed. Such trend can be related to the decreasing number of phenolic –OH groups in individual main cannabinoids and to the interaction of OH groups with electrons of double bond occurring in the non-olivetolic fragments of these compounds [19]. In consequence, the ability of electron transport from the cannabinoid molecules to the ABTS cation radical is diminished and/or hindered in the mentioned order. As the trend is more visible for NAT than for ART and MONO oils (*F*_exp_NAT > *F*_exp_ART and *F*_exp_MONO), the influence of the antagonistic and synergistic interactions between the cannabinoids and other components of hemp extracts present in the former oils on the intensity of this trend cannot be excluded.
- The obtained results indicate that the differences in the antioxidant activity of oils with acidic and neutral cannabinoids, both prepared from the plant extracts and obtained in the synthetic way, are statistically insignificant (*F*_exp_ < *F*_crit_) with the exception of THCA and THC oils for which (*F*_exp_ > *F*_crit_) [individual (*F*_exp_) values are collected in section E of Table 3]. According to the literature [20], the introduction of a carboxyl group to the aromatic ring of the phenolic group causes the charge delocalization and the reduction of the electron density, facilitating the phenolic radical formation. Hence, oils with the acidic forms of cannabinoids should exhibit a greater antioxidant activity than those with neutral forms. The obtained results are in contradiction with this statement. However, it should be remembered that the electron transfer from the antioxidant molecule to the ABTS cation radical is accompanied by proton detachment from phenolic –OH group (SET mechanism). In the acidic forms of cannabinoids, the active phenolic –OH group and the –COOH group are set in the -*ortho* position. In consequence, they are involved in the formation of intramolecular hydrogen bonding. This hinders the detachment of the proton from the phenolic group. In consequence, the lack of the –COOH group influence on the antioxidant activity of some oils containing acid cannabinoids compared to those with neutral cannabinoids is observed. As THCA possesses only one phenolic OH group which is involved in the formation of intramolecular hydrogen bonding with the COOH group, an evidently smaller antioxidant activity of THCA oils compared to the THC ones is observed.

**Table 3:** F values obtained during at statistical analysis antioxidant properties of the tested cannabinoid oils

Section	Name of tested group	*F*_exp_	*F*_crit_
A	CBGA-NAT/TROLOX	1253.83	5.32
	CBDA-NAT/TROLOX	1139.90	
	THCA-NAT/TROLOX	473.45	
	CBGA-ART/TROLOX	102.23	
	CBDA-ART/TROLOX	33.44	
	THCA-ART/TROLOX	58.58	
	CBGA-MONO/TROLOX	51.13	
	CBDA-MONO/TROLOX	29.22	
	THCA-MONO/TROLOX	125.00	
B	CBGA-ART/CBGA-MONO	4.29	5.32
	CBDA-ART/CBDA-MONO	0.15	
	THCA-ART/THCA-MONO	3.38	
	CBG-ART/CBG-MONO	1.88	
	CBD-ART/CBD-MONO	0.60	
	THC-ART/THC-MONO	1.65	
C	CBGA-NAT/CBGA-ART	898.69	5.32
	CBGA-NAT/CBGA-MONO	1008.38	
	CBDA-NAT/CBDA-ART	936.21	
	CBDA-NAT/CBDA-MONO	949.93	
	THCA-NAT/THCA-ART	748.17	
	THCA-NAT/THCA-MONO	881.84	
	CBG-NAT/CBG-ART	828.94	
	CBG-NAT/CBG-MONO	928.03	
	CBD-NAT/CBD-ART	774.92	
	CBD-NAT/CBD-MONO	831.03	
	THC-NAT/THC-ART	570.92	
	THC-NAT/THC-MONO	662.80	
D	CBGA-NAT/CBDA-NAT/THCA-NAT	218.15	3.89
	CBGA-ART/CBDA-ART/THCA-ART	148.22	
	CBGA-MONO/CBDA-MONO/THCA-MONO	167.70	
	CBG-NAT/CBD-NAT/THC-NAT	124.43	
	CBG-ART/CBD-ART/THC-ART	42.02	
	CBG-MONO/CBD-MONO/THC-MONO	43.27	
E	CBGA-NAT/CBG-NAT	0.82	5.32
	CBDA-NAT/CBD-NAT	0.48	
	THCA-NAT/THC-NAT	68.80	
	CBGA-ART/CBG-ART	2.50	
	CBDA-ART/CBD-ART	5.09	
	THCA-ART/THC-ART	167.76	
	CBGA-MONO/CBG-MONO	2.88	
	CBDA-MONO/CBD-MONO	3.43	
	THCA-MONO/THC-MONO	195.21	

**Fig. 2:**
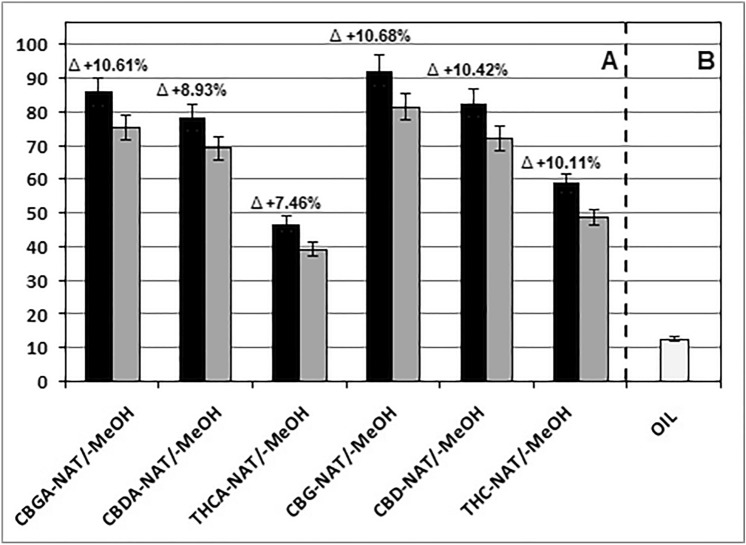
Antioxidant activity of: CBGA-NAT, CBDA-NAT, THCA-NAT, CBG-NAT, CBD-NAT and THC-NAT cannabinoid oils (black bars in Fig. 2A) and corresponding hemp plant extracts in methanol, CBGA-MeOH, CBDA-MeOH, THCA-MeOH, CBG-MeOH, CBD-MeOH and THC-MeOH (dark gray bars in Fig. 2A). The light gray bar in Fig. 2B corresponds to the antioxidant activity of hemp oil which was used as the base of the cannabinoid oils being examined

Although the base of cannabinoid oils can be any edible oil, this role is most often played by hemp oil. It should be noted that the oil base, particularly the unrefined one, also has an antioxidant activity and may influence the antioxidant activity of cannabinoid oil. This is confirmed in Fig. 2A showing the antioxidant activity of the individual hemp extracts (see gray bars for MeOH solutions) versus the antioxidant activity of the corresponding NAT oils (black bars), which were shown in this figure again for easier data comparison. It should be emphasized that the concentration of the cannabinoid dominating in the given hemp extract and NAT oil in the measuring systems with the ABTS radical cation was the same and amounted to 0.0001 M. Figure 2B shows an antioxidant activity of the hemp oil, which acted as a base in all tested oils. When determining the hemp oil antioxidant activity, its amount in the measurement system was the same as that of hemp oil during the determination of the antioxidant activity of NAT, ART and MONO cannabinoid oils. The data presented in Fig. 2 indicate the additivity of the antioxidant properties of hemp extracts and hemp oil. The difference between the mean value of antioxidant activity of a given cannabinoid oil and the corresponding hemp extract is more or less equal (within error limits) to the antioxidant activity of the hemp oil itself (*F*_exp_ = 4.85 < *F*_crit_ = 4.97).

## Conclusion

In the wide range of products containing hemp ingredients, cannabinoid oils are the most common. This results from positive effects of some cannabinoids in the treatment and prophylaxis of a wide variety of oxidation-associated diseases [4–6], but also from their ability to block SARS-CoV-2 virus from entering human cells [21]. As shown by the research, the antioxidant activity of cannabinoid oils depends on both type of the cannabinoid which dominates in the oil and the method of their preparation. Cannabinoid oils obtained from the plant extracts have the greatest antioxidant activity. In addition to cannabinoids, they also contain other plant matrix components that affect their antioxidant activity of these oils. Considering the type of cannabinoid dominating in individual oils, those containing CBG, CBGA, CBD and CBDA show greater antioxidant activity than the oils with THCA and THC. Due to the psychoactive activity of THC, the latter are approved for the use only in inpatient medicine in many countries. Recently, more and more attention has been paid to the therapeutic properties of cannabinoid oils in which the main ingredient is the acid form of a given cannabinoid. The presented results prove that the antioxidant activity of the oil with a neutral or acidic form of a given cannabinoid is similar.

## Data Availability

Not applicable.
